# Analysis of the Literature on Chronic Cough in Children

**DOI:** 10.2174/1874306401711010001

**Published:** 2017-04-27

**Authors:** Marcello Bergamini, Ahmad Kantar, Renato Cutrera, Italian Pediatric Cough Interest Group

**Affiliations:** 1 Local Health Trust of Ferrara, Ferrara, Italy; 2 Pediatric Cough and Asthma Centre, University and Research Hospitals, Istituti Ospedalieri Bergamaschi, Bergamo, Italy; 3 Respiratory Unit, Bambino Gesù Children's Hospital, Rome, Italy; 4 Italian Pediatric Cough Interest Group: F. Antonelli (Department of Pediatric Pneumology, Santobono-Pausilipon Hospital, Naples, Italy), A. Barbato (Pediatrics Department, University of Padua, Padua, Italy), R. Bernardini (Pediatric Unit, San Giuseppe Hospital, Empoli, Italy), E. Bignamini (Pneumology Unit, AOU Città della Salute e della Scienza, Turin, Italy), F. Cardinale (Pediatric Unit, Division of Pulmonology, Allergy and Immunology, AOU “Policlinico-Giovanni XXIII”, Bari, Italy), S. Cazzato (Department of Pediatrics, University of Bologna, St. Orsola-Malpighi Hospital, Bologna, Italy), M. Ghezzi (Pediatric Pulmonary and Allergy Unit, Istituto Giannina Gaslini, Genoa, Italy), F. Midulla (Department of Pediatric Emergency, “Sapienza” University, Rome, Italy), M. Miraglia del Giudice (Department of Pediatrics, Second University of Naples, Naples, Italy), A. Novelli (Clinical Pharmacology and Oncology Section, University of Florence, Florence, Italy), V. Ragazzo (Pediatric Unit, San Giuseppe Hospital, Empoli, Italy), G.A. Rossi (Pediatric Pulmonary and Allergy Unit, Istituto Giannina Gaslini, Genoa, Italy), O. Sacco (Pediatric Pulmonary and Allergy Unit, Istituto Giannina Gaslini, Genoa, Italy), A. Saggin (School of Management, Bocconi University, Milan, Italy), B. Tagliaferri (Department of Radiology, Melloni University Hospital, Milan, Italy), G. Tancredi (Department of Pediatrics, “Sapienza” University, Rome, Italy), L. Terracciano (Department of Pediatrics, Melloni University Hospital, Milan, Italy), N. Ullmann (Respiratory Unit, Bambino Gesù Children's Hospital, Rome, Italy), A. Zanasi (Pneumology Unit, University of Bologna, S. Orsola Malpighi Hospital, Bologna, Italy).

**Keywords:** Chronic cough, Children, Guidelines, Cough management

## Abstract

Throughout childhood, various developmental phenomena influence the cough reflex. Among these are the modifications in the anatomy and functions of the respiratory tract and the central and peripheral nervous systems. Moreover, after birth, the immunological response undergoes progressive transformations with the acquisition of immune memory processes. These conditions make infections and airway abnormalities the overwhelming cause of chronic cough in children and infants. In children, chronic cough should be treated on the basis of etiology. The aim of this article is to provide thorough research and analysis of the medical literature published up to 2014 on chronic cough in children as a disease entity, including the epidemiologic, etiologic, diagnostic, prognostic, and therapeutic aspects.

Our results demonstrate differences in the definition of chronic cough, the characteristics of diagnostic procedures, study settings, and prevalence of the main causes. However, few studies regarding epidemiology and the quality of life have been reported. Many therapeutic approaches that are considered effective in adults with chronic cough seem to be less efficient in children. Regardless of the setting, whether pediatric or non-pediatric, children with chronic cough should be carefully evaluated using child-specific protocols and algorithms. Awareness of the various pathophysiological conditions associated with chronic cough is vital for making a correct diagnosis and providing appropriate treatment. The prevalence of the different causes of chronic cough depends on various issues. Among these are the population under consideration and its age range, infectious disease control and prevention, the diagnostic procedures employed, disease definition criteria, and the local health system. Clinical guidelines for the management of children with chronic cough should take these components into consideration. Further clinical and basic research studies are still needed for better diagnosis, treatment, and prevention of chronic cough in children.

## INTRODUCTION

The cough reflex causes modifications of normal breathing patterns [1]. This reflex is a complex and precisely timed neuromuscular phenomenon that involves the diaphragm, chest wall muscles, cervical muscles, abdominal muscles, laryngeal abductor and adductor muscles, and both medullary and cortical brain regions [2-4]. Throughout childhood, various developmental phenomena influence the cough reflex. Among these are modifications in the anatomy and functions of the respiratory airways and the central and peripheral nervous systems. Furthermore, because of the immature immunological response, infection is the main cause for cough in infancy. In general, children are more vulnerable to environmental factors. The impairment or absence of the cough reflex can be harmful and even fatal in some disease conditions. However, a cough may be the first symptom of many diseases or conditions affecting the respiratory tract, as a cough can represent more than a defense mechanism. Through its persistence, it becomes a helpful indicator of potential disease conditions for both patients and physicians. Almost all diseases of the respiratory tract, and in some cases of the extra-respiratory tract, can cause chronic cough. For the physician, it is most important to identify the serious disorders that require prompt management [5].

The aim of this article is to provide thorough research and analysis of the existing medical literature up to 2014 on chronic cough in children as a disease entity, including its epidemiologic, etiologic, diagnostic, prognostic, and therapeutic aspects. Given that chronic cough is not commonly considered a concrete pathological entity, but rather a persistent symptom of a chronic disease, reports in the literature addressing a single disorder causing chronic cough were not included in our present research.

## RESEARCH METHODOLOGY

The following sources were selected to identify the primary studies, secondary sources, and guideline searches:

- MedLine (through the PubMed search engine)
- EMBASE
- Cochrane Database of Systematic Reviews (CDSR; The Cochrane Library)
- Database of Abstract of Reviews of Effects (DARE)
- The principal Guideline Banks (LG) on the website of Gruppo Italiano di Medicina Basata sulle Evidenze (GIMBE) [6].

From the keyword registers in Embase (Emtree) and PubMed (MeSH database), we employed the following terms for data collection: ‘cough’, ‘coughing’, and ‘chronic’, matched case-by-case in separate search strings to the keywords ‘epidemiology’, ‘prevalence’, ‘incidence’, ‘etiology’, ‘diagnosis’, ‘prognosis’, and ‘therapy’. In PubMed, we used the pre-defined phrase ‘chronic cough children’ and the search tool for specific clinical areas known as clinical queries.

With these, it was possible to separately explore the fields of etiology, diagnosis, prognosis and therapy, as well as any ‘clinical prediction guides’ that were available. Our literature search was conducted without time limits and was concluded on September 8, 2014. The selection was further restricted to reports written in English or Italian and involving humans within the age range of 0 to 18 years. For articles regarding therapy, we limited our search to randomized controlled trials (RCTs). In our investigation, we excluded non-systematic reviews, editorials, letters, works of pure research, and grey literature.

## RESULTS AND DISCUSSION

### Prevalence Studies

There are limited data reporting the prevalence of chronic cough in children. ln a study conducted in 2002 on a population of 2275 children aged 1-15 years living in rural areas of India, chronic recurrent cough was present in 1.06% of cases [7]. Whereas in a study conducted in China in 2010 on children living in six different urban areas, a 21-28% increase in chronic cough was observed for each interquartile increase in the concentration of atmospheric pollutants, such as total suspended particles, nitrogen dioxide, and sulphur dioxide [8].

Data from an Australian prevalence study [9] that was conducted on a cohort of children enlisted following urgent admittance to a tertiary center for acute respiratory disease reported that 20% of the children (aged 1-14 years old) had chronic cough (cough for more than 4 weeks). However, this percentage represents the prevalence in a selected population that was admitted to a tertiary center following acute respiratory illness, which is quite different from that in the general population.

More pertinent to Italian settings was the SIDRIA-2 observational study [10] that was conducted on 33,000 children and adolescents in various parts of the country. This study showed a significant increase in cough and chronic catarrh with an increase in prevalence from 2.2% in traffic-free areas to 3.2% in areas with intense traffic. Moreover, this study demonstrated differences between areas with rare truck passage (prevalence 2.0%) and areas with frequent (2.9%) or continuous (3.9%) truck passage. The increase in the prevalence of chronic cough associated with low automobile traffic was not significant.

### Diagnosis

Bibliographic research in terms of EBM revealed only one strictly diagnostic publication, which was an Australian study conducted in 2006 [11]. The prospective cohort study included 100 children with an average age of 2.8 years who coughed significantly and persistently for more than 3 weeks. Although the trial’s overall methodological quality was good, it is worth keeping in mind that these children had a median cough duration of 6 months, suggesting a possible high level of disease severity.

The authors identified wet cough as a good indicator of a specific cough (a cough with a specific cause), which was the primary aim of the study. In reality, however, with a positive likelihood ratio (LR+) barely above 1 (1.29), this parameter seems to provide an irrelevant diagnostic performance in both populations. As expected, better positive predictability was provided by hemoptysis and chronic dyspnea. In addition, the study revealed diagnostic tools with a positive predictability for a specific etiology, which included the following: objective chest examination with an LR+ of 2.4, radiography anomalies with an LR+ of 2.92, and spirometry with an LR+ of 2.33. This latter examination was performed in only 32 children who were older than 6 years of age. Wet cough was shown to be the lone parameter that was effective mainly in excluding a specific cause in a low-prevalence population (LR- of 0.15). However, factors including history (mainly pointers), clinical evaluation, and diagnostic tools each had an LR- >0.3, thus, demonstrating the scarcity of the efficacy in an exclusion diagnosis.

Based on these results, the authors subsequently recommended in their guidelines chest radiography and spirometry as diagnostic tools that should be used in children who have experienced cough for longer than 4 weeks.

### Cough Questionnaire

A recent systematic review [12] determined that only the pediatric cough quality of life (PC-QoL) questionnaire can be considered a valid means for assessing the severity and impact of a cough on a child’s health and that evidence on the validity of cough diaries or visual analogue scale scores is currently insufficient. Four relevant publications were obtained using PubMed clinical queries. Two of these publications focused on the validation of the PC-QoL [13, 14], one was dedicated to the validation of a questionnaire on the quality of the cough [15], and the remaining publication regarded the validation of a constructed questionnaire on cough hypersensitivity syndrome [16] that was not pertinent to the scope of this review.

### Etiology and Algorithms for Management of Chronic Cough

The earliest publication we found in this field was a systematic review (SR) on the causal relationship between parental smoking and various respiratory symptoms in children, including cough [17]. The review, which included 34 studies, demonstrated that the odds ratio (OR) for exposure to parental smoking and cough was 1.40 (95% confidence interval [CI] 1.27-1.53). The authors concluded that the relationship between parental smoking and respiratory symptoms “seems very likely” to be causal given the statistical significance, robustness to adjustment for confounding factors, consistency of the findings in different countries, and evidence of dose response.

In the above-cited observational study that was conducted in a rural Indian population of 2275 children, Singh *et al.* reported bronchial asthma as the most common cause of chronic cough in 66.7% of the studied group, followed by post-nasal drip in 25%. A family history of smoking was recognized in 16.7% of cases, which is in contrast to 6.4% in the control group (p=0.05). There was no significant association with overcrowding, presence of pets, or the category of fuel employed for home cooking. However, this study cannot be compared to case studies in Western nations because of the large differences in health services, hygiene, and socioeconomic conditions.

The second study by the Italian SIDRIA-2 Group was conducted in 2005 and focused on indoor exposure and respiratory and allergic disorders [18]. The study revealed that pollution was a significant causal link between chronic cough, exposure to domestic molds, and the presence of dogs during the first year of life (OR 1.89; 95%CI 1.31-2.71 and OR 1.87; 95%CI 1.12-3.11, respectively). Indoor exposure to dogs in the first year of life apparently mantains its effects even throughout adolescence (OR 2.03; 95%CI 1.12-3.67). The last four publications found during our research were conducted in different international settings and with more rigorous bases. The earliest of these was a study performed by an Australian group over a 2-year period. In that study, 108 children with an average age of 2.8 years and chronic cough were subjected to a standardized sequence of a diagnostic pathway approach in which chest radiography and spirometry were primarily performed when possible. Bronchoscopic investigation, computed tomography scanning, and oesophageal pH-metric monitoring were included in the second phase of the pathway [19].

The second report is a Turkish study published in 2008 that was conducted on 108 children with an average age of 8.5 years that applied the recommendations encompassed in the guidelines of the American College of Chest Physicians (ACCP) [20, 21]. In that study, children with specific cough alarm indicators (pointers) were excluded from the algorithm at the beginning. In a third prospective study carried out in the USA that lasted 4 years, a fairly small number of children (n=40) with an average age of 7.8 years and persistent cough for more than 8 weeks were subjected to a rather intensive and detailed work-up that is typical of a third-level center [22]. The fourth publication involved a larger cohort than those in the previous three studies. It included 346 children from six Australian centers (five centers were located in cities, and one center was located in a rural area) [23]. Moreover, children were separated into four age groups (0-2, 2-6, 6-12, and >12 years) and were managed in accordance with a pathway similar to the one utilized by the Turkish authors that followed the ACCP guidelines in which chest radiography and spirometry were conducted during an early phase. In the absence of specific pointers, the children underwent a diagnostic-therapeutic trial with inhaled corticosteroids when the chronic cough was dry/unproductive or with antibiotics when the cough was wet/productive.

As can be observed in a summary of these studies in Table **1**, large differences are evident, including differences in the definition of the chronic cough duration (4 versus 8 weeks), age groups, number of subjects, and duration of the cough prior to enrolment (16 weeks in the studies by Asilsoy and Chang, 18 weeks in the study by Khoshoo, and 6 months in the study by Marchant). Moreover, the study by Khoshoo differs from the other 3 studies by the lack of an a priori definition of the ascribed etiology and how much change was considered to be important. Differences were observed in the-characteristics of the adopted diagnostic procedures, the settings in which the studies were conducted, the initial inclusion or exclusion criteria (children presenting alarm signals indicating a cough with specific etiology were excluded in Asilsoy’s study) and in the methodological quality of statistical analyses.

The four above-mentioned studies present noteworthy differences in the frequencies of various etiologic diagnoses, which were highest for asthma, gastroesophageal reflux (GER), and upper airway disorders (post-nasal drip) in the older populations (Khoshoo and Asilsoy studies). In the younger populations studied, the diagnosis with the highest frequency was protracted bacterial bronchitis. The significance of this latter correlation was demonstrated in the Australian multi-center study in which protracted bacterial bronchitis was observed in approximately 54% of children less than 2 years old (n=124), in 40% of children aged between 2 and 4 years (n=126), in 27% of children aged between 6 and 12 years (n=82), and in 29% of those older than 12 years (n=14). No differences or trends were evident in the frequencies of the other four most often-reported diagnoses. However, a significantly higher frequency of tracheomalacia was observed in the aborigine population than in the white population (29.4% vs. 6.7%; p=0.001).

### Intervention Studies

To assess the validity of management according to the standardized clinical management, we examined a single recent Australian trial study [24, 25]. That study is the only study dedicated to the use of algorithms subsequent to the 2009 Cochrane SR that failed to identify any pertinent RCT [26]. The multi-center study used a pragmatic design to test whether management of chronic cough in children in accordance with an evidence-based pathway was feasible, reliable, and improved clinical outcomes. The principal objective was to test the efficacy of the pathway. Because an RCT comparing use versus non-use of the pathway was not possible, the authors designed their study to evaluate delayed versus early employment of the pathway. Approximately 75% of the group of children enrolled in an Australian trial [23] had previously been members of a population examined in a randomized study [24, 25]. The intervention consisted of the use of a diagnostic-therapeutic algorithm at different timings: 2-3 weeks after the request for admission (early-arm) or 5-7 weeks after the request for admission (delayed-arm). One hundred and forty children with an average age of 4.6 years were randomly selected to undergo intervention within 2-3 weeks, whereas 132 received the intervention within 5-7 weeks, which represented the standard caregiving time at the various centers that were involved in the study. The aim of the study was to demonstrate, with an 82% power and a 5% significance level, the statistical significance of a difference of at least 20% in the “resolution of the cough” within 6 weeks of acceptance for admission (this parameter constituted one of the two primary outcomes of the randomized controlled trial). The other primary outcome was the difference in the parent-proxy cough-specific quality of life (PC-QoL) completed within 6 weeks of acceptance for admission. At the end of the study, the intention-to-treat analysis demonstrated a statistically significant difference for the resolution of the cough within 6 weeks (54.3% vs. 29.5%; p=0.01; number needed to treat [NNT]=4). The authors also affirmed that a significant improvement in PC-QoL was observed in both groups and was greater in the early treated group (the p-value was not specified). That study, in which blindness was obviously not applicable, had good methodological quality in terms of other risks of potential bias. The period of the cough duration in randomized patients was fairly long (4 months), which must be noted particularly in comparison with the Italian children, for whom access to pediatric care is easier, thus, allowing earlier initiation of treatment.

### Chronic Cough Treatment

An examination of the secondary sources not included in the Cochrane Collaboration revealed a review lacking the minimum systematic criteria that we decided not to include [27] and an SR that was conducted in 2013 [28] in which childhood studies constituted only a scarcely relevant part of the overall study. The CDSR instead presents an “umbrella review” composed of up to 15 SRs that are continuously updated on the website, which provides an important, complete, and methodologically irreprehensible body of evidence. However, it currently lacks wide clinical significance because of the general scarcity of well-structured studies on pharmacological and non-pharmacological therapies for chronic cough in children. One of these 15 SRs examined the management algorithms that were mentioned earlier in this review [26]. Five SRs [29-33] did not include any pertinent work, nor did our research reveal more recent pertinent trials. These include trials with chromones, anticholinergic agents, obstructive sleep apnea syndrome therapies, modification of indoor environments, and honey or cough drop usage. Regarding β-2 agonists, no efficacy was demonstrated by the only RCT study that was included in a dedicated SR [34]. The same applies to the two trials included in an SR examining the use of leukotriene receptor antagonists [35]. In an SR examining the use of methylxanthine, some cohort studies appeared to have demonstrated an efficacy of dubious clinical relevance on the symptoms of chronic cough despite the well-known undesired side-effects of the drug [36]. An SR on the use of antibiotics in a chronic cough post-bronchiolitis indicated only one RCT in which the efficacy of clarithromycin was not significant [37].

An SR on antihistamines included three therapeutic studies with conflicting results. The data from the two larger therapeutic studies that described no beneficial effect are in contrast to a smaller study that demonstrated that the second-generation antihistamine cetirizine was beneficial in reducing coughs associated with seasonal allergic rhinitis [38].

In regard to these latter Cochrane SR references [29-33], our research failed to discover any subsequent noteworthy trials. There are two SRs regarding the use of inhaled corticosteroids [39, 40]. Among the various selected works, only one trial (which contained serious methodological defects) demonstrated a relatively modest efficacy of fluticasone dipropionate for nocturnal cough [41]. The drug was administered at a high dosage of 2 mg/day for 3 days, then 500 μg/day for the following 11 days. None of the dietetic or pharmacological treatments studied in the six RCTs of an SR examining the treatment of GER were shown to be effective [42]. However, it is also worth noting that in the work conducted with proton pump inhibitors, only 1 patient of the 11 that were treated experienced an undesired side effect (number needed to harm = 11) [43]. Only one work was published after the SR on GER by Chang *et al.*; this work was a Chinese RCT conducted on 19 children assigned to four groups in which the omeprazole-bethanecol combination appeared to reduce daytime coughing in children with GER [44].

The SR on the use of antibiotic therapy [45] instead selected only two randomized trials of modest quality: one conducted with erythromycin and the other with an under-dose of amoxicillin clavulanate. In the authors’ opinion, on the basis of only these two studies, it would be necessary to treat three children (NNT=3) with an antibiotic in order to obtain a significant improvement in persistent cough. These conclusions were conditioned by the methodological weakness of the study. One particularly relevant study was published after the final update of the latter SR, which was an RCT conducted in Australia in which 50 children with an average age of 1.9 years and with wet cough lasting for more than 3 weeks were randomized to 2 weeks of twice daily oral amoxicillin clavulanate (22.5 mg/kg/dose) or placebo [46]. In this selected population, which was treated with low antibiotic doses (higher doses are commonly used in Italy), the NNT was only three children (95%CI 1.8-13.9).

### Guidelines

We consider that there are four main international guidelines (American, British, Belgian, and Australian) on chronic cough in children that are most relevant [21, 47-49], all of which are important documents because they were produced by research groups of cough experts. Most of these groups have studied the phenomenon in great detail over the past two decades. Although numerous articles published in recent years have modified our knowledge of the etiology and management (algorithms and therapy) of chronic cough, until now (September 2014), none of the guidelines have been updated or revised. Moreover, the qualitative analysis of the three fundamental characteristics according to which guidelines should be produced (multidisciplinarity, systematic literature searches, and recommendation grading) leads to appraisals that often cannot be judged positively.

The ACCP guidelines were published in 2006; this version was not multidisciplinary (only physicians were involved), and the bibliographic assessment of the methodological criteria is not easy to consult [21].

The Belgian guidelines are not multidisciplinary, and they employed a non-conventional method of recommendations [47]. The British guidelines lack multidisciplinarity, and bibliographic research was performed by the first author alone and was restricted to articles in English [48].

The Australian guidelines are the most recently updated guidelines [49]. The authors state their recommendations according to the GRADE approach. Nonetheless, the authors referred to an inaccessible online document for details regarding the methodology. In addition to this, the authors declare that the research source was limited to PubMed. Only the British Thoracic Society guidelines offer a clear correlation between single recommendations and the sources of evidence supporting them. Although all these guidelines contain practical flow-charts for a diagnostic and/or therapeutic approach for children with chronic cough, they all had a restricted panel of reviewers. Moreover, the limited number of studies and investigations in this field has the potential to introduce bias. An updated cough guideline and an expert panel is expected to be published in the coming months by an international group [50].

## CONCLUSION

Chronic cough is common in the pediatric population, yet the true prevalence of this condition remains difficult to define. Before receiving appropriate management, many children with chronic cough and their families continue to experience unnecessary or recurring medical consultation with a great impact on their quality of life. Physiologically, there are similarities and also significant differences between adults and children [51, 52]. As expected, the etiologies and management of cough in children differ from those used for adults. A chronic cough in a child should be treated on the basis of etiology; no evidence supports the use of medication for symptomatic cough relief. The benefits to the child of making an accurate diagnosis and prescribing adequate treatment, therefore, extends well beyond merely eliminating the cough.

Regardless of the setting, whether pediatric or non-pediatric, children with chronic cough should be carefully evaluated using child-specific protocols and algorithms. Those based on good evidence are more likely to improve the clinical outcomes. Knowledge of the pathophysiology of the different conditions that cause chronic cough is essential for disease management. Distinguishing the different phenotypes is helpful in achieving the correct diagnosis. There is a continuing search for the distinctive phenotypes of chronic cough patients who will prove responsive to one treatment rather than another [46]. The prevalence of each etiology in a medical setting depends on various issues. Among these are the population under consideration and the variety in the ages of its constituents (infants, preschool, school-children), infectious disease control and prevention, diagnostic procedures employed, disease definition criteria, and the health system. Clinical algorithms for the management of children with chronic cough should consider these factors. Despite the apparently high prevalence of cough in children, the topic has been poorly researched. Further clinical and basic research studies are still needed for better diagnosis, treatment, and prevention of chronic cough in children.

## Figures and Tables

**Table 1:** Principal causes of chronic cough in children.

	PBB	Asthma	UACS	GER	Bronchiectasis	Tracheo-malacia	Habitual-psychogenic cough	Spontaneous resolution	Other
*Marchant 2006* [19] (2.6 yrs average age)	40%	4%	3%	3%	6%	-	1%	22%	21%
*Asilsoy* *2008* [20] (8.4 yrs average age)	23%	25%	20%	5%	3%	-	4%	6%	3%
*Khoshoo 2009* [22] (7.8 yrs average age)	-	13%	23%	28%	-	-	10%	-	25%
*Chang* *2012* [23] (4.5 yrs average age)	41%	15.8%	1.4%	2.3%	9%	6.1%	4.3%	13.9%	6.1%

Abbreviations: PBB: protracted bacterial bronchitis; GER: gastroesophageal reflux; UACS: upper airway cough syndrome
